# Concurrent Nivolumab-Induced Gastritis and Cholangitis Accompanied by Biliary Tract Hemorrhage in a Patient With Stage IV Lung Adenocarcinoma

**DOI:** 10.7759/cureus.46392

**Published:** 2023-10-02

**Authors:** Akihiro Tsukaguchi, Norihiko Yamaguchi, Hiroyuki Ogawa, Toshiyuki Ikeda

**Affiliations:** 1 Department of Respiratory Medicine, Nishinomiya Municipal Central Hospital, Nishinomiya, JPN; 2 Department of Gastroenterology, Nishinomiya Municipal Central Hospital, Nishinomiya, JPN

**Keywords:** lung adenocarcinoma, biliary tract hemorrhage, nivolumab, immune-related cholangitis, immune-related gastritis

## Abstract

Immune checkpoint inhibitors, including nivolumab, can result in immune-related adverse events (irAEs) that may affect multiple organ systems. Among irAEs, both gastritis and cholangitis are uncommon. We present the case of a 65-year-old man who received nivolumab for lung adenocarcinoma presented with epigastric pain. He was diagnosed with immune-related gastritis and cholangitis based on imaging and pathological findings. We administered prednisolone (1 mg/kg/day), which improved the patient’s gastritis and relieved his pain. However, he experienced recurrent epigastric pain during corticosteroid tapering, and magnetic resonance imaging showed biliary tract hemorrhage. After watchful waiting, the hemorrhage improved without additional immunosuppressants. Immune-related gastritis, immune-related cholangitis, and their coexistence should be considered in patients who develop epigastric pain during immune checkpoint inhibitor therapy. When patients with concurrent immune-related gastritis and cholangitis complain of recurrent epigastric pain, it is important to assess which of these two irAEs is worsening because the optimal immunosuppressants differ between the two.

## Introduction

Immune checkpoint inhibitors (ICIs) have remarkably improved the prognosis of many types of cancer. However, the increased use of ICIs, including nivolumab, has also increased the incidence of immune-related adverse events (irAEs), which can affect multiple organ systems [1]. Immune-related gastritis manifests as diffuse gastritis with erythematous and edematous mucosa, and histologic examination reveals epithelial denudation with severe inflammatory cell infiltration [2]. Immune-related cholangitis is characterized by biliary dilatation with diffuse hypertrophy of the extrahepatic biliary tract wall, and a pathological feature is a cluster of differentiation (CD)3-positive lymphocyte infiltration especially around Glisson’s capsule [3,4]. In previous studies, immune-related gastritis and cholangitis were observed in only 1.1% and 0.8% of patients treated with ICIs, respectively [2,5]. Only two cases of coexisting gastritis and cholangitis have been reported. In this study, we report a case involving a patient with concurrent immune-related gastritis and cholangitis accompanied by biliary tract hemorrhage.

## Case presentation

Male in the seventh decade of life with a smoking history of 44 pack-years, currently a former smoker with no other significant history, was referred for treatment of pleural effusion and a lung tumor in the right hilar region. The result of brushing and bronchoscopic biopsy was conclusive of advanced stage lung adenocarcinoma in brushing cytology since the biopsy was negative. Extrathoracic staging, bone and liver metastatic lesions were found. The tumor proportion score for programmed death-ligand 1 (PD-L1) was high (≥50%) as assessed using the PD-L1 IHC 22C3 pharmDx kit (Agilent Technologies, Santa Clara, CA, USA) in the cytologic cell block sample of bronchial brushing specimens. Platinum doublet chemotherapy treatment was started due to the lack of molecular alterations for targeted therapy. Upon evaluation of the tumor response by RECIST 1.1, disease progression was documented after four cycles, so a second-line scheme with nivolumab was started. The initial dose of nivolumab was 240 mg every two weeks. The dosing schedule of nivolumab was changed to 480 mg every four weeks after two cycles. We continued nivolumab treatment because whole-body CT revealed that the primary lesion and the mediastinal lymph node and liver metastatic lesions had shrunk after three cycles of nivolumab.

**Figure 1:**
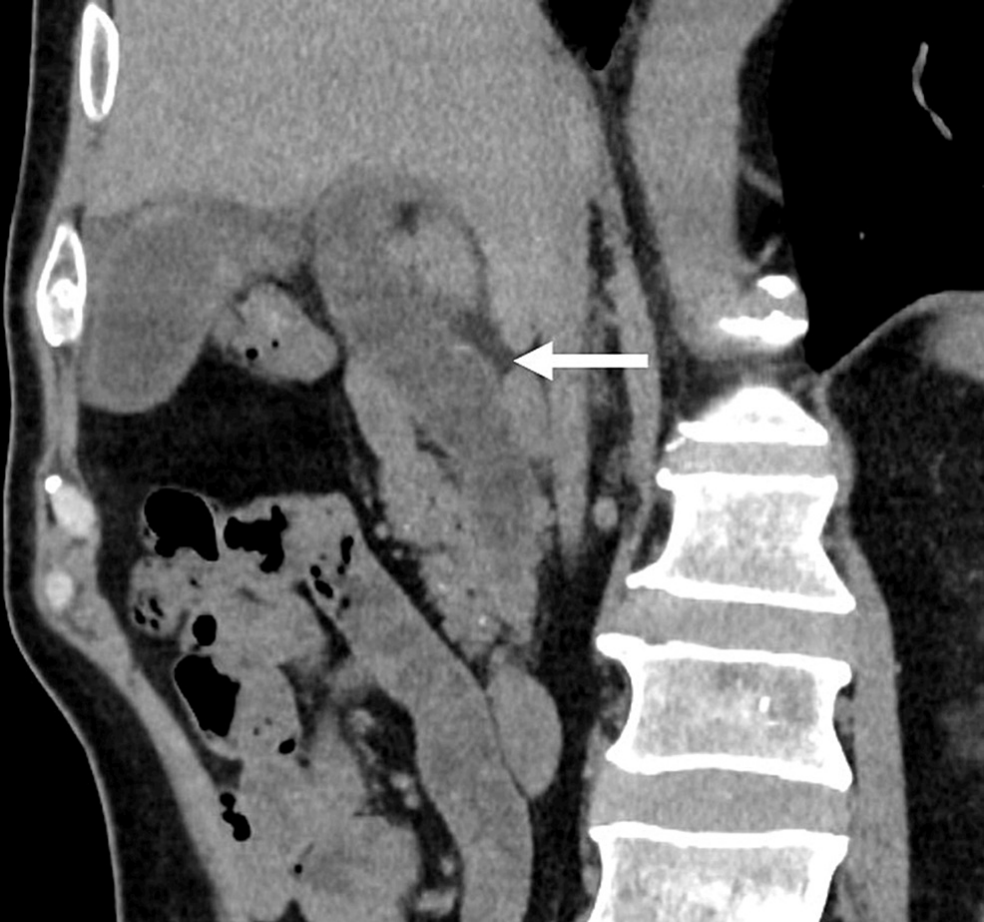
Abdominal computed tomography on admission. Remarkable dilatation and wall thickening of the extrahepatic bile duct (arrow) were noted.

After receiving eight cycles of nivolumab, the patient complained of epigastric pain. At 218 days after nivolumab initiation and 39 days after the last dose, he presented to the emergency department with persistent epigastric pain and anorexia. Blood examination revealed elevated levels of alanine aminotransferase (ALT) (46 U/L), alkaline phosphatase (ALP) (126 U/L), and gamma-glutamyl transpeptidase (GGT) (104 U/L), all of which were categorized as grade 1 in the Common Terminology Criteria for Adverse Events. The aspartate aminotransaminase (AST) level, total bilirubin level, prothrombin time, and platelet count were within the normal ranges. CT revealed remarkable dilatation and wall thickening of the extrahepatic bile duct and edematous thickening of the gallbladder wall (Figure 1). There was no evidence of progression of liver metastasis. Magnetic resonance cholangiopancreatography revealed dilatation of the extrahepatic bile duct without obstruction, and there were no tumors, debris, or gallstones.

**Figure 2:**
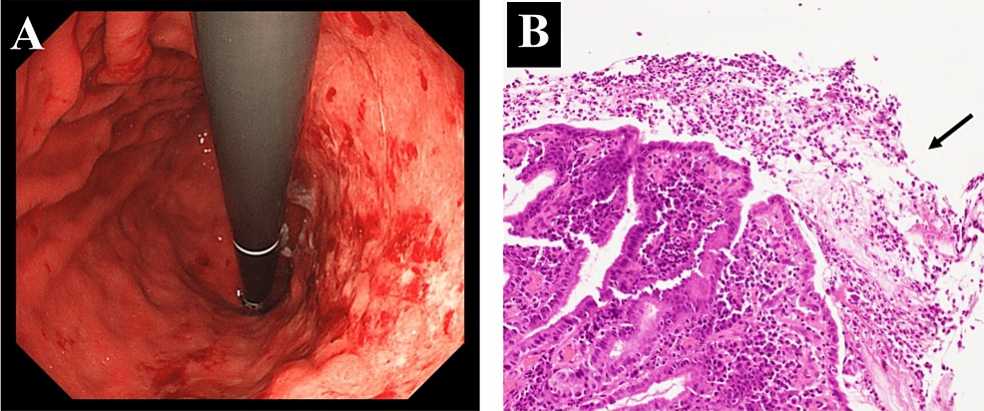
(A) Esophagogastroduodenoscopy before corticosteroid treatment and (B) histopathological examination of gastric mucosa specimens. Epithelial denudation and fibroinflammatory exudate on the epithelia were noted (arrow).

On day 4 of hospitalization, we performed esophagogastroduodenoscopy to exclude a tumor of the ampulla of Vater. Endoscopy showed that the gastric mucosa was erythematous and edematous with diffuse, irregular erosions (Figure 2A). Histological examination of biopsies from the antrum and corpus of the stomach revealed epithelial denudation with severe inflammatory (lymphocytic and plasmacytic) cell infiltration in the glandular epithelium and fibroinflammatory exudate on the epithelia (Figure 2B). There was no evidence of malignancy, cytomegalovirus infection, or Helicobacter pylori infection. We diagnosed the patient with grade 3 nivolumab-induced gastritis based on the endoscopic and pathological findings.

**Figure 3:**
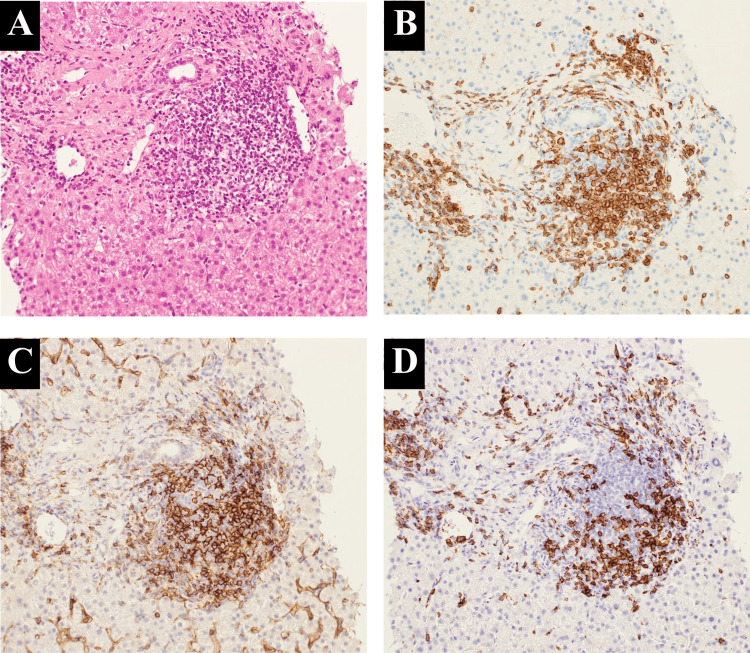
Histopathological examination of the liver specimens. (A) Hematoxylin-eosin staining (×200) and immunostaining (×200) for (B) CD3+, (C) CD4+, and (D) CD8+ lymphocytes were performed. CD3-positive lymphocyte infiltration was present especially around Glisson’s capsule, where both CD4-positive and CD8-positive lymphocytes were observed. CD, cluster of differentiation.

Because endoscopy did not reveal a tumor of the ampulla of Vater, we performed serological screening to identify the cause of hepatobiliary enzyme elevation. Blood examination revealed no active viral hepatitis infection; however, evidence of previous cytomegalovirus and Epstein-Barr virus infection was found. Although antinuclear antibody was positive at 1:160 dilution and the antimitochondrial M2 antibody level was higher than normal at 23.2 U/mL (normal range, 0-7.0 U/mL), the serum levels of IgM, IgG, and IgG4 were within the normal ranges. Therefore, we performed liver biopsy on day 14 of hospitalization to clarify the cause of the injury. Histological and immunohistochemical examination revealed CD3-positive lymphocyte infiltration, especially around Glisson’s capsule, where both CD4-positive and CD8-positive lymphocytes were observed (Figures 3A-3D). There was no evidence of chronic non-suppurative destructive cholangitis or granuloma formation, as is observed in primary biliary cholangitis. Unlike autoimmune hepatitis, neither interface hepatitis nor CD138-positive plasma cell infiltration was conspicuous. Considering the radiologic and pathological findings, we made a diagnosis of immune-related cholangitis.

**Figure 4:**
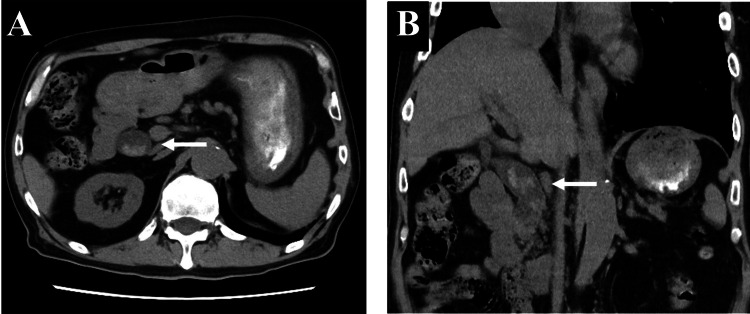
Abdominal computed tomography after the recurrence of epigastric pain. A high-density lesion was detected in the extended extrahepatic bile duct (arrow) in the (A) axial and (B) coronal sections.

**Figure 5:**
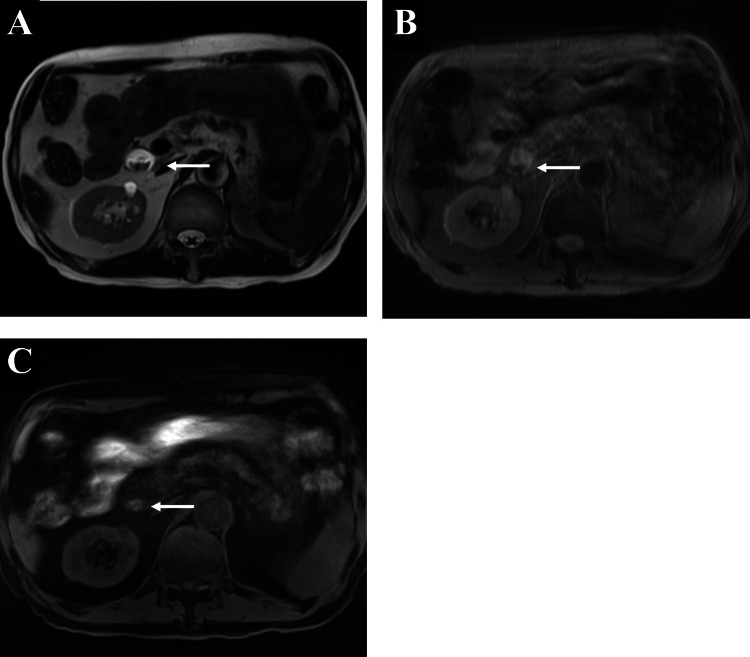
Magnetic resonance imaging after the recurrence of epigastric pain. The lesion in the extrahepatic bile duct (arrow) had low signal intensity on (A) T2- and (B) T2 star-weighted images and intermediate to high signal intensity on (C) T1-weighted images.

We introduced prednisolone (PSL) (1 mg/kg/day) for immune-related gastritis and cholangitis on day 15 of hospitalization. After PSL initiation, the patient’s gastric mucosa improved on endoscopic images, and his epigastric pain was relieved. We confirmed the absence of relapse of gastritis on the endoscopic images on days 26 and 49 of hospitalization. There was no remarkable increase in hepatobiliary enzyme levels, and a CT scan on day 45 of hospitalization showed persistent dilatation, but improved wall thickening of the extrahepatic bile duct. Based on laboratory, endoscopic and radiologic examination, we gradually tapered the PSL dose. However, he developed epigastric pain again at night on day 62 of hospitalization while receiving PSL at 20 mg/day. The laboratory analysis revealed increases in the levels of AST (218 U/L; grade 3), ALT (264 U/L; grade 3), ALP (210 U/L; grade 1), and GGT (324 U/L; grade 2). Abdominal CT showed a high-density lesion in the extended extrahepatic bile duct (Figures 4A, 4B). On magnetic resonance imaging, the lesion had low signal intensity on T2- and T2 star-weighted images and intermediate to high signal intensity on T1-weighted images, suggesting deoxyhemoglobin formation consistent with acute or subacute hemorrhage (Figures 5A-5C).

**Figure 6:**
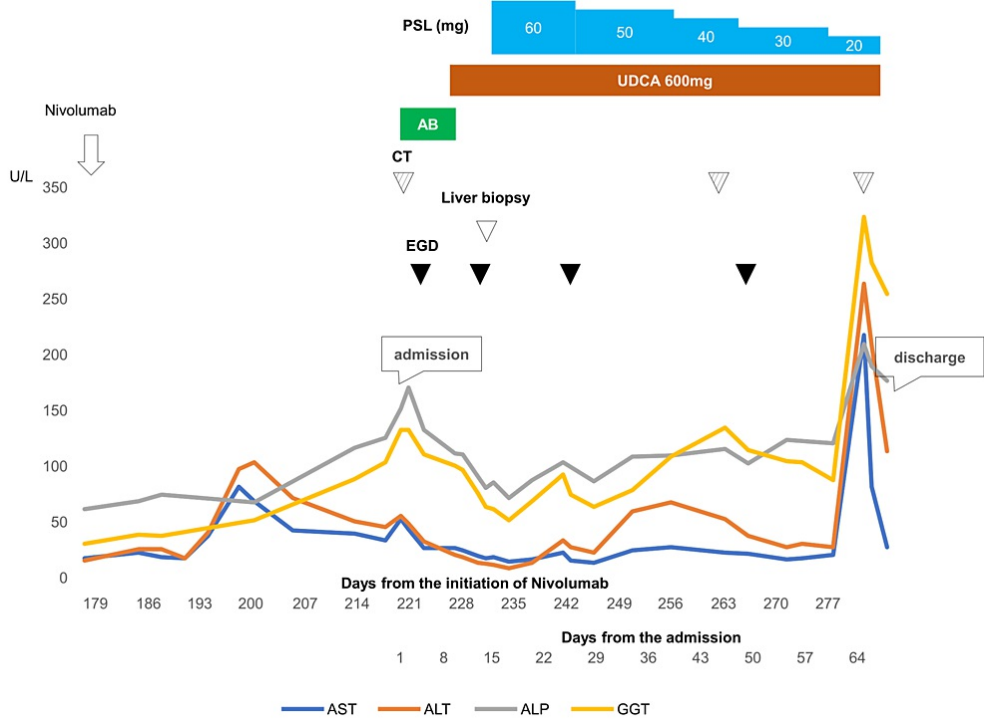
Clinical course and changes in laboratory data after administration of eight cycles of nivolumab. AB, antibiotics; ALP, alkaline phosphatase; ALT, alanine transaminase; AST, aspartate aminotransferase; CT, computed tomography; EGD, esophagogastroduodenoscopy; GGT, gamma-glutamyl transpeptidase; PSL, prednisolone; UDCA, ursodeoxycholic acid.

Because his symptom had already improved spontaneously, we considered temporary biliary tract hemorrhage. Given the additive effect of high-dose corticosteroid on the risk of infection when introducing cytotoxic chemotherapy after the progression of lung cancer, we chose close observation without increasing the corticosteroid dose. His hepatobiliary enzyme levels gradually decreased without additional immunosuppressants, and there was no evidence of hematoma expansion on follow-up abdominal ultrasound (Figure 6).

However, his primary lung cancer lesion progressed, and a new metastasis developed in the left cerebrum. We then introduced docetaxel (60 mg/m^2^ on day 1) every three weeks as the third-line regimen approximately 11 months after treatment initiation and approximately five months after the last dose of nivolumab.

## Discussion

We have herein described a patient who developed concurrent nivolumab-induced gastritis and cholangitis accompanied by biliary tract hemorrhage. To our knowledge, only three cases of coexisting gastritis and cholangitis, including our case, have been described to date (Table 1) [6,7]. All three patients presented with abdominal pain as the initial symptom. The duration from the initiation of ICI treatment to the onset of concurrent irAEs widely ranged from three to 13 months. Therefore, it is important for clinicians to be aware that immune-related gastritis and cholangitis have varied times of onset and to avoid delays in diagnosis. When patients receiving ICIs complain of abdominal pain, immune-related gastritis, and cholangitis should be listed as differential diagnoses, and their coexistence should be considered as another possibility.

**Table 1:** Summary of the present and previous cases of coexisting immune-related gastritis and cholangitis. F, female; ICI, immune checkpoint inhibitor; IV, intravenous; M, male; mPSL, methylprednisolone; PO, per oral; PSL, prednisolone

Authors, year	Age Sex	Cancer type	ICI	Time to symptom onset	Symptoms	Initial treatment	Follow-up
Cǎlugǎreanu et al., 2019 [6]	43, F	Melanoma	Nivolumab	13 months	Epigastric pain, Anorexia	mPSL 1 mg/kg IV	Cholangitis developed under PSL (1 mg/kg PO), but both gastritis and cholangitis improved after 8 weeks of treatment
Fujii et al., 2020 [7]	50s, M	Unknown primary undifferentiated cancer	Atezolizumab	3 months	Abdominal pain, General fatigue	PSL 0.6 mg/kg PO	Both gastritis and cholangitis improved
Our case	65, M	Lung adenocarcinoma	Nivolumab	7 months	Epigastric pain	PSL 1 mg/kg IV	Despite pain relief and improvement of gastritis, biliary tract hemorrhage occurred

In the present case, the causality assessment for adverse drug reaction described by Naranjo algorithm was seven, indicating that nivolumab was the probable cause of gastritis and cholangitis [8]. Furthermore, in our case, magnetic resonance imaging showed biliary tract hemorrhage during corticosteroid tapering. It was reported that CD8-positive T-cells infiltrated the biliary epithelium of the patients with immune-related cholangitis and this might induce an intense immune response leading to biliary tract hemorrhage. In this case, multiple scarred lesions were found in the extrahepatic bile ducts [9]. Cholangioscopy revealed ulcerative lesions with “burned-out” epithelium of the extrahepatic bile duct, which was erosive and easily bleeding in another case [10]. Based on the present and previous reports, biliary tract hemorrhage can accompany immune-related cholangitis. The present case is rare with respect to the coexistence of immune-related gastritis and cholangitis and the presence of secondary biliary tract hemorrhage.

Our patient complained of recurrent epigastric pain during corticosteroid tapering. Immune-related gastritis and cholangitis are treated by discontinuing the ICI and starting corticosteroids, commonly methylprednisolone or prednisone at 1 to 2 mg/kg/day [11,12]. However, cholangitis exhibits a poorer response to corticosteroid therapy than does gastritis [2,3,12,13]. Additional immunosuppressants are commonly used in refractory cases of these irAEs [13-15]. Although the types of immunosuppressants differ between immune-related gastritis and cholangitis. The use of tumor necrosis factor-α blockers is recommended for immune-related gastritis [1]. However, azathioprine or mycophenolate mofetil is reportedly used in most cases of refractory immune-related cholangitis [14,15]. The absence of reports of cholangitis treated with tumor necrosis factor-α blockers may be due to liver toxicity. Because the suitable immunosuppressants differ in corticosteroid-refractory cases, it is important to determine which of the two conditions (immune-related gastritis or cholangitis) is worsening.

During the corticosteroid tapering phase, esophagogastroduodenoscopy and abdominal ultrasound are important because these imaging examinations are useful for early assessment of deteriorated and severe cases of gastritis and cholangitis [2,16]. The corticosteroid tapering schedule for these irAEs has not been established. However, tapering corticosteroid should be done cautiously because it was reported that histopathological findings still showed residual inflammation despite an improvement in the patients’ symptoms and in the imaging findings after three months of corticosteroid treatment [17,18]. Moreover, in the previous case, combined immunosuppressive therapy with PSL, mycophenolate mofetil and tacrolimus did not improve cholangitis and the patient died of liver failure [16]. Considering that immune-related cholangitis has a long-lasting inflammation and poor response to corticosteroid, and can be life-threatening even with combined immunosuppressive therapy, we would not recommend rechallenging ICIs.

A limitation of this study is that we had only a three-month observation period after the appearance of biliary tract hemorrhage. Therefore, we had no further information on the long-term prognosis of cholangitis.

## Conclusions

We present a case involving a patient with concurrent immune-related gastritis and cholangitis accompanied by biliary tract hemorrhage. Immune-related gastritis, immune-related cholangitis, and their coexistence should be considered in patients with epigastric pain during ICI therapy. When patients with these concurrent irAEs develop recurrent epigastric pain during corticosteroid tapering, it may be important to assess which irAE is worsening to ensure selection of the optimal treatment.
